# Brain natriuretic peptide precursor (NT-pro-BNP) levels predict for clinical benefit to sunitinib treatment in patients with metastatic renal cell carcinoma

**DOI:** 10.1186/1471-2407-10-489

**Published:** 2010-09-14

**Authors:** Konstantinos T Papazisis, Lukas F Kontovinis, Christos N Papandreou, George Kouvatseas, Christos Lafaras, Evangelos Antonakis, Maria Christopoulou, Charalambos Andreadis, Despoina Mouratidou, Alexandros H Kortsaris

**Affiliations:** 1 3rd Department of Medical Oncology, Theagenion Cancer Hospital, Al. Simeonidi str. 2, 54007, Thessaloniki, Greece; 2 Applied Molecular Oncology Laboratory, Theagenion Cancer Hospital, Al. Simeonidi str. 2, 54007, Thessaloniki, Greece; 3 Department of Medical Oncology, University Hospital of Larissa, Biopolis 41110, Larissa, Greece; 4 Health Data Specialists Ltd, 11526, Athens, Greece; 5 Cardiology Unit, Theagenion Cancer Hospital, Al. Simeonidi str. 2, 54007, Thessaloniki, Greece; 6 Laboratory of Biochemistry, Theagenion Cancer Hospital, Al. Simeonidi str. 2, 54007, Thessaloniki, Greece; 7 Laboratory of Biochemistry, Department of Medicine, Democritus University of Thrace, Alexandroupolis, Greece

## Abstract

**Background:**

Sunitinib is an oral, multitargeted tyrosine kinase inhibitor that has been approved for the treatment of metastatic renal cell carcinoma. Although the majority of sunitinib-treated patients receive a clinical benefit, almost a third of the patients will not respond. Currently there is no available marker that can predict for response in these patients.

**Methods:**

We estimated the plasma levels of NT-pro-BNP (the N-terminal precursor of brain natriuretic peptide) in 36 patients that were treated with sunitinib for metastatic clear-cell renal carcinoma.

**Results:**

From the 36 patients, 9 had progressive disease and 27 obtained a clinical benefit (objective response or disease stabilization). Increases in plasma NT-pro-BNP were strongly correlated to clinical outcome. Patients with disease progression increased plasma BNP at statistically significant higher levels than patients that obtained a clinical benefit, and this was evident from the first 15 days of treatment (a three-fold increase in patients with progressive disease compared to stable NT-pro-BNP levels in patients with clinical benefit, p < 0.0001). Median progression-free survival was 12.0 months in patients with less than 1.5 fold increases (n = 22) and 3.9 months in patients with more than 1.5 fold increases in plasma NT-pro-BNP (n = 13) (log-rank test, p = 0.001).

**Conclusions:**

This is the first time that a potential "surrogate marker" has been reported with such a clear correlation to clinical benefit at an early time of treatment. Due to the relative small number of accessed patients, this observation needs to be further addressed on larger cohorts. More analyses, including multivariate analyses are needed before such an observation can be used in clinical practice.

## Background

Sunitinib is an oral, multitargeted tyrosine kinase inhibitor of VEGFR-1, VEGFR-2, Fms-like tyrosine kinase receptor 3 (FLT3), c-KIT (stem-cell factor [SCF] receptor) and PDGFR [1] that has shown clinical activity in the treatment of metastatic renal cell carcinoma. The majority of sunitinib-treated renal cancer patients obtain a clinical benefit in the form of either objective response or disease stabilization (31% and 48% respectively in the large, phase III trial [2]). Treatment with sunitinib results in alterations of several plasma proangiogenic factors, as sVEGFR2, VEGF, PDGF and PIGF [3]. However, none of these markers has a predictive value for response and the search for a "surrogate marker" is still ongoing [4].

Cardiac toxicity is reported to be a common adverse event in sunitinib-treated patients [5]. Schmidinger et al, reported that from 86 renal cancer patients that were treated with sunitinib or sorafenib, 33.6% experienced a cardiac event, 40.5% had ECG changes and 18% were symptomatic [6]. On a report by Rixe and colleagues, patients that developed hypertension during treatment (or other sunitinib-related adverse events) had a better outcome than normotensive ones [7], but this observation remains to be confirmed. Brain natriuretic peptide (BNP) has been shown to be associated with left ventricular dysfunction [8]. This peptide is secreted by atrial and/or ventricular myocardial cells due to volume or pressure overload, according to the severity of myocardial dysfunction. Pro-BNP, the precursor of BNP may provide the same diagnostic information as BNP, but less is known about its clinical utility. Brain natriuretic peptide (BNP) and N-terminal (NT) pro-BNP are useful as diagnostic objective markers of chronic heart failure (CHF) due to systolic and diastolic dysfunction and important prognostic predictors [9].

## Methods

We recently reported on a cohort of patients treated with sunitinib for metastatic clear-cell renal carcinoma in our department [10]. Following-up these data we estimated the plasma levels of NT-pro-BNP in 36 sunitinib-treated patients as a marker for cardiac toxicity. Sunitinib was administrated in the usual scheme (50 mg daily, four weeks on treatment, followed by two weeks off treatment, on a six - week cycle). During the first two cycles, plasma was obtained every 2 weeks with a complete blood count and a full biochemical profile. All patients had an appropriate renal, liver, cardiac and bone marrow function [10]. The plasma levels of NT-pro-BNP were analyzed using Elecsys 2010 automated immunoassay analyzer. The study was approved by the local ethics review board (Theagenion Cancer Hospital Research and Ethics Committee) and was carried out in accordance with the Declaration of Helsinki and Good Clinical Practice Guidelines. All patients had confirmed metastatic disease (CT and/or MRI and bone scan), were informed for their participation and signed the appropriate consent form.

### Statistical Analysis

The Chi-square test was used for testing significance of correlation for categorical variables. Univariate comparisons of Plasma NT-pro-BNP levels as well as changes from baseline where performed with the Wilcoxon non-parametric test. Logistic regression analysis was used in order to assess jointly the effects of baseline Plasma NT-pro-BNP levels and day 15 changes on disease progression. The area under the Receiver Operating Characteristic (ROC) curve is also reported. Time to event curves were calculated with the Kaplan-Meier method. Indicative cutpoint determination in PFS analysis was made using the method of Contal and O'Quigley (1999) [11] but there was no formal assessment of the significance of the cutpoint due to the small size of the sample.

## Results

Data on the patient cohort are presented on table 1. At the end of cycle 2, patients were assessed for response. Patients that obtained a clinical benefit (CB, disease stabilization or objective response according to the RECIST criteria) remained on treatment, whilst patients with progressive disease (PD) were discontinued. From the 36 patients, 9 had progressive disease and 27 obtained a clinical benefit (11 had disease stabilization and 16 had partial responses). Development of hypertension during the first two cycles did not predict for response (37.5% in the PD group and 40.74% in the CB group, p = 0.8).

**Table 1:** Patient characteristics

Patient characteristics		%
**Number**		
Total included	36	100
Assessable for response	36	100

**Sex**		
Male	26	72
Female	10	28

**Age**		
Median	62	
Range	25-75	

**Performance status**		
0	16	44.4
1	16	44.4
2	4	11.1

**MSKCC criteria**		
Low risk	2	5.6
Intermediate risk	15	41.7
High risk	19	48.7

**Previous treatments**		
Nephrectomy	30	83.3
Cytokines	15	41.7
Radiotherapy	10	27.8

**Metastatic sites**		
Lung	24	66.7
Bone	10	27.8
Lymph nodes	7	19.4
Liver	4	11.1
Local relapse	2	5.6
Other	5	13.9

**Figure 1:**
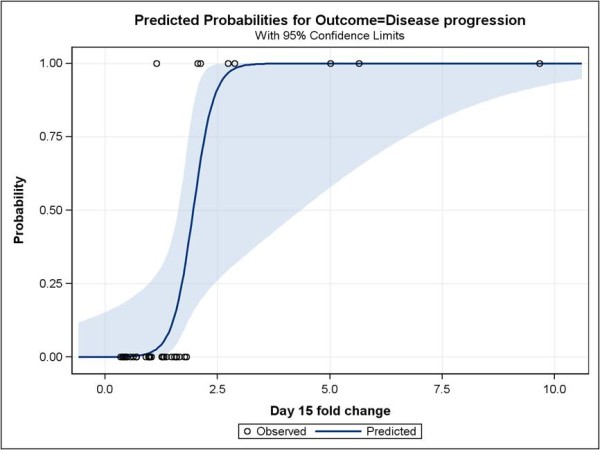
**Predicted probability of disease progression according to fold-increase in plasma NT-pro-BNP after two weeks of sunitinib treatment**. The effect of increase is statistically significant (p-value = 0.0274) but the 95% confidence limits of predicted probability are wide due to the small size of the sample (shaded area).

Plasma NT-pro-BNP was increased during treatment and did not return to baseline values during the off-treatment period. This increase was not associated to changes in arterial pressure or decreased left ventricular ejection fraction, as it was assessed by echocardiogram or MUGA test. Baseline values as well as increases in plasma NT-pro-BNP were strongly correlated to clinical outcome. Patients with disease progression had lower baseline values and increased plasma BNP at statistically significant higher levels than patients that obtained a clinical benefit (table 2), and this was evident from the first 15 days of treatment. Using logistic regression we assessed the joint effect of baseline values and change at 15 days on disease progression in order to depict the most significant predictor. Since in the logistic regression model baseline NT-pro-BNP levels were not statistically significant while change at 15 days was (p-value = 0.2912 and 0.0274 respectively) it appears that the latter has most of the information in separating the two groups. Regression to the mean might have influenced this result. Furthermore, the area under the ROC curve for the change at 15 days was 94.9%, while the high correlation between change at 15 days and disease progression is also depicted in figure 1 through the sharp increase in predicted probability of disease progression when there is an increase in plasma NT-pro-BNP levels.

**Table 2:** Median and first and third quartile levels (pg/ml) and medianfold-increase in plasma NT-pro-BNP in patients receiving sunitinib according to clinical outcome after the first two cycles of treatment.

	Baseline			Actual values after baseline								Median fold change to baseline			
								
				Day15		Day30		Day45		Day75					
**Clinical outcome**	**N**	**Median**	**Q1-Q3**	**Median**	**Q1-Q3**	**Median**	**Q1-Q3**	**Median**	**Q1-Q3**	**Median**	**Q1-Q3**	**Day15**	**Day30**	**Day45**	**Day75**
Disease progression	9	61.1	39.5-120.4	132.3	75.7-341.2	145.8	96.6-553.3	576.8	54.5-827.0	344.0	191.9-1071.0	2.8	3.9	8.9	5.4
Clinical benefit	27	149.0	97.7-535.6	179.5	80.5-337.5	215.2	87.4-380.6	241.7	111.2-555.5	260.1	120.6-637.4	1.0	1.0	0.9	0.9
p-value	0.021			0.768		0.951		0.726		0.374		0.0002	0.002	0.003	0.025

Note: Q1 and Q3 are the first and third quartiles respectively

Median PFS was 6.9 months in patients (n = 15) with increases in plasma NT-pro-BNP after 15 days of treatment and 15.8 months in patients (n = 20) where plasma NT-pro-BNP remained at the baseline or lower values (p = 0.036, log-rank test).

**Figure 2:**
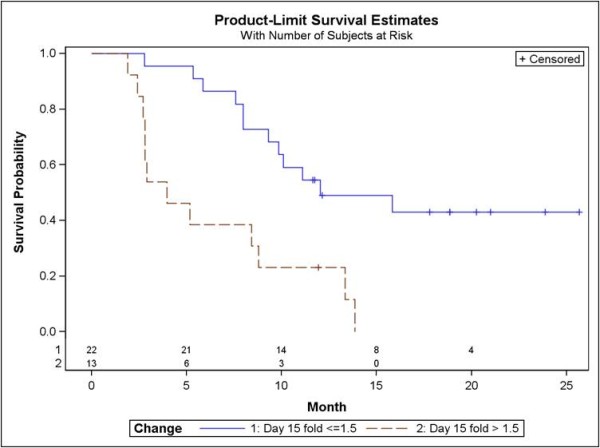
**Progression-free-survival in metastatic renal cell carcinoma patients according to fold-increase in plasma NT-pro-BNP after two weeks of sunitinib treatment**. Median PFS was 12.0 months in patients with less than 1.5 fold increases or lower or equal to baseline NT-pro-BNP levels (n = 22) and 3.9 months in patients with more than 1.5 fold increases in plasma NT-pro-BNP (n = 13) (log-rank test, p = 0.001).

We further explored the effect of change at 15 days on PFS in order to select with a statistical technique an alternative cutpoint for grouping the patients into separate groups. By testing several alternatives, the maximum value of the log-rank statistic was obtained at the 1.5 fold change. Median PFS (figure 2) was 3.9 months in patients (n = 13) with more than 1.5 fold increases in plasma NT-pro-BNP after 15 days of treatment and 12.0 months in patients (n = 22) where plasma NT-pro-BNP showed less than 1.5 fold increases or remained at the baseline or lower values (p = 0.001, log-rank test).

NT-pro-BNP levels in healthy population are depended on age and gender [12]. In our cohort of metastatic RCC patients, NT-pro-BNP levels were not statistically significantly different in men than women and in older (> 60 years old) that younger patients. Age and gender had no impact on the differences observed in fold-increase of NT-pro-BNP between PD and CB patients (Additional file 1: Table S1).

## Discussion and Conclusions

Plasma NT-pro-BNP levels may reflect volume overload or cardiac dysfunction in patients with heart disease. We observed a high variation in pre-treatment NT-pro-BNP levels that was not associated to disease burden or baseline cardiac function. However, Baseline levels predicted for response and sharp increases in plasma NT-pro-BNP were observed in patients that did not obtain any clinical benefit from treatment, and this was evident from the first 15 days of treatment. BNP is produced by myocardial cells, but it has been reported to be produced by many tissues including renal parenchyma as well [13]. Whether this observation reflects the increasing production of NT-pro-BNP from the non-responding metastatic foci remains yet unanswered. When we assayed Caki-1 and Caki-2 renal cancer cell lines *in vitro *we did not find any significant BNP production and sunitinib had no effect of on BNP at a protein level (data not shown).

On the other hand, hypoxia in large treatment-refractory tumours is a direct and sufficient stimulus for BNP induction via stabilization of HIF-1 alpha [14]. Sunitinib treatment results in improved blood flow and less hypoxic tumour microenviroment [15,16] in patients that respond. Disease progression can therefore relate to a more hypoxic tumour environment, higher HIF-1 alpha expression, higher plasma VEGF levels (as we have already shown, ref 10) and, finally, increased HIF-1 alpha-dependent BNP induction.

We report the value of plasma NT-pro-BNP measurement for predicting response to sunitinib treatment in patients with metastatic renal cell carcinoma. This is the first time that a potential "surrogate marker" has been reported with such a clear correlation to clinical benefit at an early time on treatment. Due to the relative small number of assessed patients, this observation needs to be further addressed on larger cohorts. More analyses, including multivariate analyses are needed before such an observation can be used in clinical practice.

## Competing interests

The authors declare that they have no competing interests.

## Authors' contributions

KTP designed the study, evaluated the patients, evaluated the results and prepared the manuscript. LFK was responsible for the treatment of the patients, collection of the data and evaluation of the results. CNP critically reviewed the manuscript and the interpretation of the data. GK performed the statistical analysis and helped with the interpretation of the data. CL performed the cardiac assessment of the patients and was involved in the preparation of the manuscript. EA and MC helped with the plasma databank and performed the immunoassay tests. CA participated in the clinical part of the study. DM supervised the project and participated in the clinical part of the study. AHK overviewed the analysis of the data and the manuscript. All authors read and approved the final manuscript.

## Pre-publication history

The pre-publication history for this paper can be accessed here:

http://www.biomedcentral.com/1471-2407/10/489/prepub

## Supplementary Material

Additional file 1 **Table S1**. Baseline plasma NT-pro-BNP levels (pg/ml) and medianfold ratio after 15 days of sunitinib treatment according to age and gender. CB = Clinical benefit, PD = Progressive Disease. Click here for file
