# Supplementary material for: Brain natriuretic peptide precursor (NT-pro-BNP) levels predict for clinical benefit to sunitinib treatment in patients with metastatic renal cell carcinoma
Source: BMC Cancer. 2010 Sep 14;10:489. doi: 10.1186/1471-2407-10-489 (PMC2946311; doi:10.1186/1471-2407-10-489)
Supplement: Additional file 1 — Table S1. Baseline plasma NT-pro-BNP levels (pg/ml) and medianfold ratio after 15 days of sunitinib treatment according to age and gender. CB = Clinical benefit, PD = Progressive Disease. [file 1471-2407-10-489-S1.DOC]

**Additional flies:**

Additional file 1

Table S1. Baseline plasma NT-pro-BNP levels (pg/ml) and medianfold ratio after 15 days of sunitinib treatment according to age and gender. CB = Clinical benefit, PD = Progressive Disease.

|  | **Baseline levels (pg/ml)** | **Ratio (day 15/0)** | **Ratio in CB patients** | **Ratio in PD patients** |
| --- | --- | --- | --- | --- |
| Men | 391.5 | 1.85 | 0.94 | 3.91 |
| Women | 195.1 | 1.19 | 1.19 |  |
| *p* | *0.40* | *0.35* | *0.21* | *ND* |
|  |  |  |  |  |
| ≤60 years age | 150.7 | 1.70 | 1.06 | 3.47 |
| > 60 years age | 504.3 | 1.67 | 1.03 | 4.84 |
| *p* | *0.10* | *0.96* | *0.88* | *0.59* |
